# Efficiency of genomic selection using Bayesian multi-marker models for traits selected to reflect a wide range of heritabilities and frequencies of detected quantitative traits loci in mice

**DOI:** 10.1186/1471-2156-13-42

**Published:** 2012-05-31

**Authors:** Dagmar NRG Kapell, Daniel Sorensen, Guosheng Su, Luc LG Janss, Cheryl J Ashworth, Rainer Roehe

**Affiliations:** 1 Sustainable Livestock Systems Group, Scottish Agricultural College, West Mains Road, Edinburgh, EH9 3JG, UK; 2 Faculty of Science and Technology, Department of Molecular Biology and Genetics, Aarhus University, DK-8830, Tjele, Denmark; 3 The Roslin Institute and R(D)SVS, The University of Edinburgh, Easter Bush, Midlothian, EH25 9RG, UK

**Keywords:** Genomic Selection, Bayesian Analysis, Heritabilities, Quantitative Trait Loci

## Abstract

**Background:**

Genomic selection uses dense single nucleotide polymorphisms (SNP) markers to predict breeding values, as compared to conventional evaluations which estimate polygenic effects based on phenotypic records and pedigree information. The objective of this study was to compare polygenic, genomic and combined polygenic-genomic models, including mixture models (labelled according to the percentage of genotyped SNP markers considered to have a substantial effect, ranging from 2.5% to 100%). The data consisted of phenotypes and SNP genotypes (10,946 SNPs) of 2,188 mice. Various growth, behavioural and physiological traits were selected for the analysis to reflect a wide range of heritabilities (0.10 to 0.74) and numbers of detected quantitative traits loci (QTL) (1 to 20) affecting those traits. The analysis included estimation of variance components and cross-validation within and between families.

**Results:**

Genomic selection showed a high predictive ability (PA) in comparison to traditional polygenic selection, especially for traits of moderate heritability and when cross-validation was between families. This occurred although the proportion of genomic variance of traits using genomic models was 22 to 33% smaller than using polygenic models. Using a 2.5% mixture genomic model, the proportion of genomic variance was 79% smaller relative to the polygenic model. Although the proportion of variance explained by the markers was reduced further when a smaller number of SNPs was assumed to have a substantial effect on the trait, PA of genomic selection for most traits was little affected. These low mixture percentages resulted in improved estimates of single SNP effects. Genomic models implemented for traits with fewer QTLs showed even lower PA than the polygenic models.

**Conclusions:**

Genomic selection generally performed better than traditional polygenic selection, especially in the context of between family cross-validation. Reducing the number of markers considered to affect the trait did not significantly change PA for most traits, particularly in the case of within family cross-validation, but increased the number of markers found to be associated with QTLs. The underlying number of QTLs affecting the trait has an effect on PA, with a smaller number of QTLs resulting in lower PA using the genomic model compared to the polygenic model.

## Background

Recently, high-density single nucleotide polymorphism (SNP) arrays for a broad range of species have been developed, including humans, mice, plant species such as barley, wheat or maize as well as major livestock species, such as cattle, pigs, sheep and chickens. In the past, selective breeding in plant and livestock species was based on phenotypic information combined with extensive pedigrees using best linear unbiased prediction. The use of high density SNP arrays opened the opportunity of using genomic information to estimate genomic breeding values for individuals [1]. Estimating a breeding value based on the genotype of an individual may provide large benefits in situations where a species has a large generation interval e.g. oil palm [2] or when the trait of interest is recorded in one sex only e.g. milk production [3]. Other traits that may benefit from genomic selection are behavioural traits in animals, which are often costly and time consuming to measure routinely e.g. aggressiveness [4].

The high cost of genotyping, especially for the high density SNP arrays, limits the extent to which routine genotyping can be implemented in practice. Additionally, many of the SNPs contribute little to the genetic variance of a trait, as was found for example for human height variation [5] or complex disease traits [6]. Moreover, statistical limitations can arise when the number of SNP effects exceeds by far the amount of phenotypic data available. For these reasons, there could be interest in reducing the number of SNPs while maintaining efficiency of selection. Costs of genotyping may be reduced by genotyping only part of the population, e.g. [7], or a two-step approach could be used to prioritize SNPs for genotyping with lower density SNP arrays, e.g. [8]. To circumvent the statistical limitations, many different approaches have been developed to reduce the number of SNP effects to be estimated, e.g. [9,10].

The aim of this study was to assess the efficiency of genomic selection using mouse data and how it is affected by a) the heritability of the trait, b) the number of QTLs affecting the trait, c) the type of trait (‘classical’ traits that are easily measurable versus behavioural traits) and d) the number of SNP markers in the model allowed to have a substantial effect. Various models are fitted (including polygenic and/or genomic effects), and cross-validation performance within and between families is compared.

## Results

### Variance components

Tables 1, 2 and 3 show estimates of the total phenotypic variances, heritabilities based on polygenic effects, proportions of variances attributed to genomic effects relative to the phenotypic variance and of the phenotypic fractions of cage variances. Estimated variance components are based on the full dataset and are presented for seven models, namely: models (1), (2) and (3), and sub-models with 10% and 2.5% of the markers assumed to be associated with a substantial effect using models (2) and (3). Results based on sub-models using mixtures of 70%, 40%, 7.5% and 5% are not presented, because they showed the same trend as the 10% and 2.5% mixtures.

**Table 1:** Estimated variance components and heritabilities for weight traits

**Trait**^**1**^	**Model**^**2**^	$\sigma^{2}{{}_{p}}^{3}$	$\sigma^{2}{{}_{c}}^{4}$	$\sigma^{2}{{}_{e}}^{5}$	$h^{2}{{}_{u}}^{6}$	$h^{2}{{}_{a}}^{7}$
	(1)	110.8 _99.5–122.8_	31.3 _24.6–38.1_	21.1 _9.4–32.5_	0.52 _0.38–0.69_	-
	(2) 100%	117.1 _108.1–126.1_	39.0 _31.5–46.4_	35.8 _32.4–39.3_	-	0.36 _0.32–0.40_
	(2) 10%	104.7 _96.3–113.6_	43.8 _36.1–51.6_	43.8 _40.0–47.5_	-	0.16 _0.12–0.21_
**W6**	(2) 2.5%	104.8 _96.1–113.4_	46.9 _38.7–55.5_	46.3 _42.5–49.9_	-	0.11 _0.07–0.15_
	(3) 100%	119.0 _107.9–129.6_	33.1 _26.2–40.5_	22.5 _13.9–30.5_	0.25 _0.12–0.36_	0.29 _0.25–0.33_
	(3) 10%	107.9 _98.1–117.7_	32.7 _25.9–39.6_	25.8 _17.5–33.2_	0.33 _0.21–0.46_	0.12 _0.08–0.16_
	(3) 2.5%	108.8 _99.0–118.8_	32.3 _25.0–38.9_	24.2 _15.7–32.4_	0.40 _0.28–0.53_	0.08 _0.05–0.11_
	(1)	110.0 _98.7–122.0_	31.5 _24.3–38.3_	22.2 _11.2–33.8_	0.51 _0.36–0.66_	-
	(2) 100%	118.0 _108.3–126.6_	39.1 _31.2–46.6_	36.1 _32.7–39.7_	-	0.36 _0.33–0.40_
	(2) 10%	103.7 _95.1–112.3_	45.9 _38.3–54.2_	46.1 _42.3–49.8_	-	0.11 _0.07–0.15_
**W6m**	(2) 2.5%	104.3 _95.5–113.3_	49.3 _40.8–57.9_	48.2 _44.4–52.0_	-	0.06 _0.03–0.10_
	(3) 100%	117.6 _107.9–128.1_	33.8 _26.8–41.3_	26.2 _19.5–32.9_	0.20 _0.10–0.31_	0.29 _0.25–0.33_
	(3) 10%	104.8 _96.5–114.7_	32.9 _26.2–40.4_	28.7 _21.6–36.5_	0.35 _0.23–0.47_	0.06 _0.03–0.10_
	(3) 2.5%	106.6 _96.7–116.1_	32.2 _25.4–39.3_	26.4 _17.6–35.1_	0.41 _0.27–0.54_	0.04 _0.01–0.07_
	(1)	125.7 _113.6–139.9_	19.0 _12.8–25.4_	36.6 _22.1–50.3_	0.55 _0.40–0.71_	-
	(2) 100%	133.8 _124.9–143.3_	28.2 _21.2–35.0_	48.2 _43.3–52.7_	-	0.43 _0.40–0.47_
	(2) 10%	120.2 _111.5–129.3_	31.6 _24.2–38.6_	58.9 _54.0–64.5_	-	0.25 _0.20–0.29_
**W10**	(2) 2.5%	119.7 _111.0–128.7_	34.6 _27.1–42.3_	62.7 _57.7–68.0_	-	0.19 _0.14–0.23_
	(3) 100%	138.3 _126.8–150.9_	22.3 _15.9–29.0_	34.1 _24.1–43.9_	0.22 _0.10–0.33_	0.37 _0.33–0.41_
	(3) 10%	124.9 _114.3–136.1_	21.2 _15.0–27.4_	39.0 _29.6–48.6_	0.32 _0.20–0.45_	0.19 _0.15–0.25_
	(3) 2.5%	125.8 _113.6–137.4_	20.3 _14.7–26.7_	37.5 _25.6–47.7_	0.40 _0.27–0.54_	0.14 _0.09–0.18_

1. Trait: W6 = Weight at week 6; W6m = Weight at week 6, missing marker genotypes were treated as 3^rd^ allele; W10 = Weight at week 10; observations of all traits were multiplied by 10^2^.

2. Model: (1) = polygenic; (2) = genomic; (3) = polygenic and genomic; with 100/10/2.5% of the markers allowed to have an effect.

3. $\sigma^{2}{}_{\text{p}}$: estimates of the total phenotypic variances.

4. $\sigma^{2}{}_{\text{c}}$: estimates of variances attributed to the cage effect.

5. $\sigma^{2}{}_{\text{e}}$: estimates of residual variances.

6. ${\text{h}}^{2}{}_{\text{u}}$: heritability based on the polygenic effect.

7. ${\text{h}}^{2}{}_{\text{a}}$: proportion of the variance attributed to the genomic effect.

The 95%-highest posterior density intervals have been presented as subscript.

**Table 2:** Estimated variance components and heritabilities for behavioural traits

**Trait**^**1**^	**Model**^**2**^	$\sigma^{2}{{}_{p}}^{3}$	$\sigma^{2}{{}_{c}}^{4}$	$\sigma^{2}{{}_{e}}^{5}$	$h^{2}{{}_{u}}^{6}$	$h^{2}{{}_{a}}^{7}$
	(1)	61.1 _56.3–66.1_	2.3 _0.2–4.5_	37.6 _32.0–43.0_	0.35 _0.23–0.46_	-
	(2) 100%	60.0 _56.4–63.6_	3.3 _1.1–5.7_	40.5 _37.3–43.8_	-	0.27 _0.24–0.30_
	(2) 10%	60.7 _56.6–64.8_	3.8 _1.3–6.1_	41.7 _38.4–45.1_	-	0.25 _0.21–0.30_
**TA**	(2) 2.5%	60.6 _56.8–64.8_	5.0 _2.3–7.6_	43.5 _40.1–46.9_	-	0.20 _0.16–0.24_
	(3) 100%	59.7 _55.6–63.7_	2.5 _0.1–4.5_	37.8 _33.5–42.3_	0.11 _0.03–0.19_	0.22 _0.18–0.25_
	(3) 10%	60.7 _56.4–65.3_	2.5 _0.2–4.5_	38.4 _34.2–42.5_	0.12 _0.04–0.20_	0.21 _0.15–0.26_
	(3) 2.5%	61.3 _56.7–66.2_	2.3 _0.3–4.5_	37.5 _32.9–42.3_	0.21 _0.10–0.31_	0.14 _0.10–0.19_
	(1)	1243.4 _1128.1–1351.9_	45.6 _2.8–94.5_	790.0 _655.5–924.6_	0.33 _0.20–0.47_	-
	(2) 100%	1222.4 _1132.2–1308.8_	82.8 _26.4–143.5_	871.3 _787.1–951.8_	-	0.22 _0.19–0.25_
	(2) 10%	1206.3 _1119.1–1303.4_	89.2 _17.1–149.6_	905.7 _822.5–993.2_	-	0.18 _0.13–0.23_
**TF**	(2) 2.5%	1206.1 _1115.6–1304.2_	112.8 _54.5–176.9_	937.9 _852.0–1024.4_	-	0.13 _0.08–0.17_
	(3) 100%	1233.0 _1133.8–1341.0_	49.5 _0.6–99.1_	794.2 _672.4–918.1_	0.15 _0.04–0.30_	0.16 _0.13–0.19_
	(3) 10%	1240.0 _1132.4–1353.1_	47.9 _1.2–97.9_	809.6 _683.6–929.0_	0.15 _0.01–0.27_	0.16 _0.10–0.22_
	(3) 2.5%	1251.4 _1147.1–1369.7_	44.2 _1.4–91.9_	807.8 _695.4–925.8_	0.20 _0.08–0.31_	0.12 _0.07–0.17_
	(1)	1289.9 _1191.1–1382.1_	94.2 _25.9–165.1_	1066.2 _961.3–1168.9_	0.10 _0.04–0.17_	-
	(2) 100%	1290.0 _1203.0–1382.9_	105.4 _40.2–170.8_	1092.0 _1000.8–1184.1_	-	0.07 _0.06–0.09_
	(2) 10%	1286.0 _1200.1–1383.4_	111.1 _49.6–177.6_	1104.0 _1007.0–1196.4_	-	0.05 _0.02–0.09_
**FB**	(2) 2.5%	1285.0 _1197.5–1383.8_	116.2 _53.1–182.8_	1114.0 _1021.1–1211.1_	-	0.04 _0.01–0.07_
	(3) 100%	1291.0 _1200.6–1385.5_	92.3 _28.8–160.5_	1072.0 _963.6–1167.8_	0.05 _0.00–0.10_	0.05 _0.04–0.07_
	(3) 10%	1295.0 _1202.9–1393.1_	92.5 _24.2–156.6_	1065.0 _961.4–1177.0_	0.07 _0.00–0.14_	0.04 _0.00–0.07_
	(3) 2.5%	1291.0 _1199.4–1393.7_	97.3 _29.6–165.6_	1077.0 _978.0–1188.2_	0.06 _0.01–0.13_	0.03 _0.00–0.06_

1. Trait: TA = Total activity in open field test (observations were multiplied by 10^-2^); TF = Time freezing during cue; FB = Fecal boli after cue (observations were multipled by 10^1^).

2. Model: (1) = polygenic; (2) = genomic; (3) = polygenic and genomic; with 100/10/2.5% of the markers allowed to have an effect.

3. $\sigma^{2}{}_{\text{p}}$: estimates of the total phenotypic variances.

4. $\sigma^{2}{}_{\text{c}}$: estimates of variances attributed to the cage effect.

5. $\sigma^{2}{}_{\text{e}}$: estimates of residual variances.

6. ${\text{h}}^{2}{}_{\text{u}}$: heritability based on the polygenic effect.

7. ${\text{h}}^{2}{}_{\text{a}}$: proportion of the variance attributed to the genomic effect.

The 95%-highest posterior density intervals have been presented as subscript.

**Table 3:** Estimated variance components and heritabilities for physiological traits

**Trait**^**1**^	**Model**^**2**^	$\sigma^{2}{{}_{p}}^{3}$	$\sigma^{2}{}_{c}$	$\sigma^{2}{}_{e}$	$h^{2}{}_{u}$	$h^{2}{}_{a}$
	(1)	212.0 _196.7–229.8_	42.3 _28.4–56.5_	148.0 _131.3–164.1_	0.10 _0.01–0.19_	-
	(2) 100%	211.1 _196.3–227.3_	44.5 _32.0–58.1_	152.5 _139.8–165.1_	-	0.07 _0.05–0.08_
	(2) 10%	210.6 _194.0–225.8_	46.3 _32.4–59.8_	154.5 _141.2–167.6_	-	0.05 _0.00–0.08_
**HC**	(2) 2.5%	210.5 _194.7–226.5_	46.8 _33.2–60.9_	155.8 _143.7–169.6_	-	0.04 _0.00–0.07_
	(3) 100%	213.7 _196.1–229.6_	40.9 _27.9–54.8_	145.4 _128.9–162.0_	0.08 _0.01–0.17_	0.05 _0.04–0.06_
	(3) 10%	212.8 _196.6–229.8_	41.6 _27.9–55.3_	147.2 _130.1–162.7_	0.08 _0.00–0.18_	0.03 _0.00–0.06_
	(3) 2.5%	212.9 _197.0–229.7_	41.0 _27.7–54.7_	146.3 _130.1–162.3_	0.10 _0.00–0.18_	0.02 _0.00–0.05_
	(1)	806.8 _743.4–873.9_	201.9 _150.4–261.2_	475.4 _413.6–534.5_	0.16 _0.07–0.26_	-
	(2) 100%	811.5 _753.5–876.4_	215.4 _162.3–272.0_	502.4 _461.6–547.2_	-	0.12 _0.09–0.14_
	(2) 10%	798.9 _737.0–860.1_	225.1 _169.7–279.2_	517.7 _476.1–562.3_	-	0.07 _0.03–0.11_
**I75**	(2) 2.5%	798.4 _736.6–858.0_	231.0 _174.1–285.9_	525.4 _485.3–569.4_	-	0.05 _0.02–0.08_
	(3) 100%	814.9 _751.4–878.1_	199.6 _147.2–257.5_	474.1 _419.6–529.8_	0.08 _0.02–0.17_	0.09 _0.07–0.11_
	(3) 10%	803.6 _739.9–867.1_	204.2 _148.9–262.2_	487.6 _429.5–544.9_	0.08 _0.00–0.17_	0.06 _0.02–0.10_
	(3) 2.5%	806.4 _741.2–869.7_	197.9 _143.2–251.3_	474.6 _413.5–535.3_	0.13 _0.03–0.22_	0.04 _0.01–0.07_

1. HC = Hematocrit percentage (observations multiplied by 10^-2^); I75 = Insulin level (observations multiplied by 10^2^).

2. Model: (1) = polygenic; (2) = genomic; (3) = polygenic and genomic; with 100/10/2.5% of the markers allowed to have an effect.

3. $\sigma^{2}{}_{\text{p}}$: estimates of the total phenotypic variances.

4. $\sigma^{2}{}_{\text{c}}$: estimates of variances attributed to the cage effect.

5. $\sigma^{2}{}_{\text{e}}$: estimates of residual variances.

6. ${\text{h}}^{2}{}_{\text{u}}$: heritability based on the polygenic effect.

7. ${\text{h}}^{2}{}_{\text{a}}$: proportion of the variance attributed to the genomic effect.

Analyses of weight traits based on model (1), using polygenic effects only, showed slightly lower heritabilities (Table 1) compared to those reported by Valdar et al. [11]. The differences are likely because of different fixed effects fitted in the models. For behavioural and physiological traits, Tables 2 and 3 show estimates of heritabilities of comparable magnitude to those reported by Valdar et al. [11]. Using model (1), phenotypic proportions of cage variances were low for the behavioural traits (4 to 7% of the total variance, Table 2) compared to weight and physiological traits (15 to 29%, Tables 1 and 3).

Phenotypic proportions of genomic variances of weight, behavioural and physiological traits using genomic model (2) were 22 to 31%, 23 to 33% and 25 to 30% lower, respectively, than those using the polygenic model (1) (Tables 1 to 3). This was compensated for by an increase in variances attributed to the cage effects and/or error effects depending on trait. Using the 2.5% mixture in genomic model (2) – i.e. 2.5% of the genotyped SNP markers assumed to have a substantial effect on the trait – the phenotypic proportions of variance of genomic effects were 65 to 79%, 43 to 61% and 60 to 69% lower than estimates of the heritability from the polygenic model (1) for weight, behavioural and physiological traits, respectively. The underestimation of variances of genomic effects compared to variances of polygenic effects may be due to incomplete linkage disequilibrium between SNPs markers and causal variants, and due to low frequencies of these causal variants [12].

In model (3), additionally fitting polygenic effects essentially captured part of the genetic variance that was not accounted for by the genomic effects. The total variance attributed to genetic effects (polygenic and genomic) was in line with the polygenic variance found in model (1). Phenotypic proportions of the variance of the genomic effects using model (3) were consistently lower than in model (2). The phenotypic proportions of the variance of the genomic effects were 33 to 44%, 37 to 52% and 44 to 50% lower for weight, behavioural and physiological traits, respectively, than the phenotypic proportions of the variance of the polygenic effects obtained from model (1). For these traits, the phenotypic proportions of the variance of the polygenic effects accounted for 40 to 48%, 31 to 50% and 50 to 80%, respectively, of the heritability estimated using model (1). The use of different mixtures in model (3) resulted in a 75 to 92%, 60 to 70% and 75 to 80% decrease in phenotypic proportions of variance of the genomic effects compared to the corresponding proportions of polygenic effects from model (1) for the respective traits. The phenotypic proportions of the variance of the polygenic effects for weight, behavioural and physiological traits accounted for 73 to 80%, 60 to 61% and 81 to 100%, respectively, of the phenotypic proportions of the variance of the polygenic effects from model (1).

Using model (3), in general, a lower mixture percentage led to a decrease in phenotypic proportions of the variance of the genomic effects and to an increase in the phenotypic proportions of variance of the polygenic effects in all traits. Comparing W6 and W6m, treating missing alleles as a separate 3^rd^ allele, resulted in small changes in proportions of the variance of the genomic effects with lower mixture percentages.

### Predictive ability

Tables 4 and 5 show the average predictive abilities (PA) based on cross-validation within (W) or between families (B). PA was calculated using ten training and validation sets, and is shown for all three models and their sub-model using different mixtures. Within family cross-validation always performed substantially better, as expected because of the higher genetic connectedness between the training and validation dataset. Within family cross-validation resulted in little change between PAs for all models for most traits, with only W6 and TA showing an increase in PA using models (2) and (3) compared to model (1).

**Table 4:** Predictive abilities for cross-validation within (W) or between (B) families for weight traits

**Trait**^**1**^	**W6**		**W6m**		**W10**	
**Model**^**2**^	**W**^**3**^	**B**^**4**^	**W**^**3**^	**B**^**4**^	**W**^**3**^	**B**^**5**^
**(1)**	0.62	0.15	0.62	0.15	0.53	0.19
**(2) 100%**	0.63	0.24	0.63	0.23	0.57	0.29
**(2) 70%**	0.65	0.26	0.65	0.26	0.58	0.31
**(2) 40%**	0.65	0.27	0.65	0.27	0.59	0.32
**(2) 10%**	0.64	0.24	0.64	0.25	0.58	0.33
**(2) 7.5%**	0.64	0.24	0.64	0.24	0.58	0.33
**(2) 5%**	0.64	0.22	0.64	0.23	0.57	0.31
**(2) 2.5%**	0.63	0.20	0.63	0.20	0.56	0.31
**(3) 100%**	0.64	0.25	0.64	0.25	0.58	0.31
**(3) 70%**	0.65	0.27	0.65	0.27	0.59	0.33
**(3) 40%**	0.65	0.27	0.65	0.27	0.59	0.34
**(3) 10%**	0.65	0.27	0.64	0.25	0.59	0.34
**(3) 7.5%**	0.65	0.26	0.64	0.25	0.59	0.34
**(3) 5%**	0.65	0.25	0.64	0.24	0.58	0.33
**(3) 2.5%**	0.64	0.24	0.63	0.23	0.57	0.31

1: Trait: W6 = Weight at week 6; W6m = Weight at week 6, missing maker genotypes were treated as 3^rd^ allele; W10 = Weight at week 10.

2. Model: (1) = polygenic; (2) = genomic; (3) = polygenic and genomic; with 100/10/2.5% of the markers allowed to have an effect.

3. W: cross-validation within families (all s.e. ≤ 0.01).

4. B: cross-validation between families (all s.e. ≤ 0.03).

5. B: cross-validation between families (all s.e. ≤ 0.04).

**Table 5:** Predictive abilities for cross-validation within (W) or between (B) families for behavioural and physiological traits

**Trait**^**1**^	**TA**		**TF**		**FB**		**HC**		**I75**	
**Model**^**2**^	**W**^**3**^	**B**^**4**^	**W**^**5**^	**B**^**6**^	**W**^**5**^	**B**^**6**^	**W**^**3**^	**B**^**7**^	**W**^**3**^	**B**^**4**^
**(1)**	0.37	0.16	0.29	−0.04	0.21	0.10	0.33	0.08	0.42	0.08
**(2) 100%**	0.43	0.34	0.31	0.19	0.22	0.11	0.33	0.05	0.42	0.13
**(2) 70%**	0.43	0.34	0.31	0.18	0.22	0.12	0.33	0.05	0.42	0.13
**(2) 40%**	0.43	0.35	0.32	0.19	0.22	0.11	0.33	0.05	0.42	0.13
**(2) 10%**	0.42	0.34	0.33	0.20	0.21	0.11	0.33	0.06	0.42	0.14
**(2) 7.5%**	0.42	0.33	0.32	0.20	0.21	0.11	0.33	0.06	0.42	0.14
**(2) 5%**	0.41	0.30	0.32	0.19	0.21	0.11	0.33	0.06	0.42	0.13
**(2) 2.5%**	0.40	0.27	0.31	0.18	0.20	0.10	0.33	0.06	0.42	0.12
**(3) 100%**	0.43	0.33	0.33	0.17	0.22	0.13	0.33	0.06	0.43	0.13
**(3) 70%**	0.43	0.34	0.33	0.17	0.22	0.12	0.33	0.05	0.43	0.13
**(3) 40%**	0.43	0.34	0.33	0.17	0.22	0.12	0.33	0.05	0.43	0.13
**(3) 10%**	0.42	0.33	0.34	0.19	0.22	0.12	0.33	0.06	0.43	0.13
**(3) 7.5%**	0.42	0.32	0.34	0.18	0.22	0.12	0.33	0.07	0.43	0.13
**(3) 5%**	0.42	0.30	0.34	0.17	0.22	0.12	0.33	0.07	0.43	0.14
**(3) 2.5%**	0.41	0.28	0.33	0.16	0.21	0.12	0.33	0.08	0.43	0.12

1. Trait: TA = Total activity in open field test; TF = Time freezing during cue; FB = Fecal boli after cue; HC = Hematocrit percentage; I75 = Insulin level.

2. Model: (1) = polygenic; (2) = genomic; (3) = polygenic and genomic; with 100/10/2.5% of the markers allowed to have an effect.

3. W: cross-validation within families (all s.e. ≤ 0.01).

4. B: cross-validation between families (all s.e. ≤ 0.03).

5. W: cross-validation within families (all s.e. ≤ 0.02).

6. B: cross-validation between families (all s.e. ≤ 0.04).

7. B: cross-validation between families (all s.e. ≤ 0.02).

In contrast, using between family cross-validation, model (1) resulted in substantially lower PA than models (2) and (3) for most traits. This was especially visible for traits with moderate to high heritabilities (e.g. for W6: PA of 0.15 vs. 0.24 or for TF: PA of −0.04 vs. 0.19 using model (1) and (2), respectively; Tables 4 and 5). For traits with low heritabilities there were little differences in PA between model (1) and the other two models.

TA was the only trait to show significant differences in PA for within as well as between family cross-validation using model (2) with different mixtures. PA was stable at first with the low mixture models, but with mixtures below 7.5%, PA decreased substantially compared to its highest value (0.27 vs. 0.35). W6 showed a similar pattern for between family cross-validation, with a drop-off for mixtures below 7.5% (from 0.27 to 0.20). Both W6 and W10 showed a trend for a decrease in PA for within family cross-validation for mixtures below 7.5% in model (2). TA was the only trait to show a tendency for a reduction in PA with a decrease in mixture percentage, for both within (from 0.43 to 0.41) and between family cross-validation (from 0.34 to 0.28) using model (3). All other traits showed no significant decrease in PA with lower mixture percentages. Different modelling of missing genotypes as used for W6m compared to W6 showed almost no difference in PA.

### Importance of individual markers

**Figure 1:**
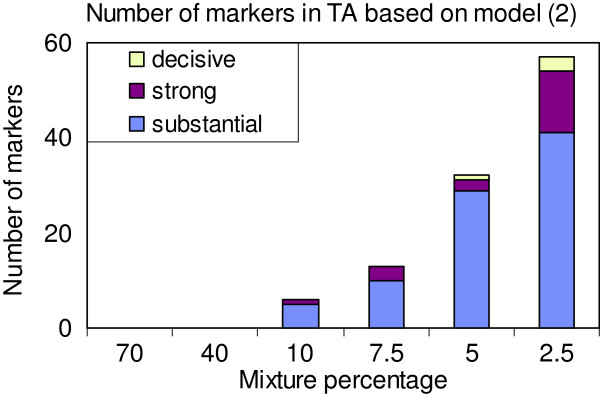
**Marker associations with QTLs based on model (2) using different mixture percentages.** Distribution of the number of SNP markers showing substantial, strong or decisive evidence to be associated with QTLs of the trait total activity in open field test (TA). Changes in odds from prior to posterior probability (PPOR) of 3.2 < PPOR ≤ 10 denotes substantial evidence, 10 < PPOR ≤ 100 strong evidence and PPOR > 100 decisive evidence.

**Figure 2:**
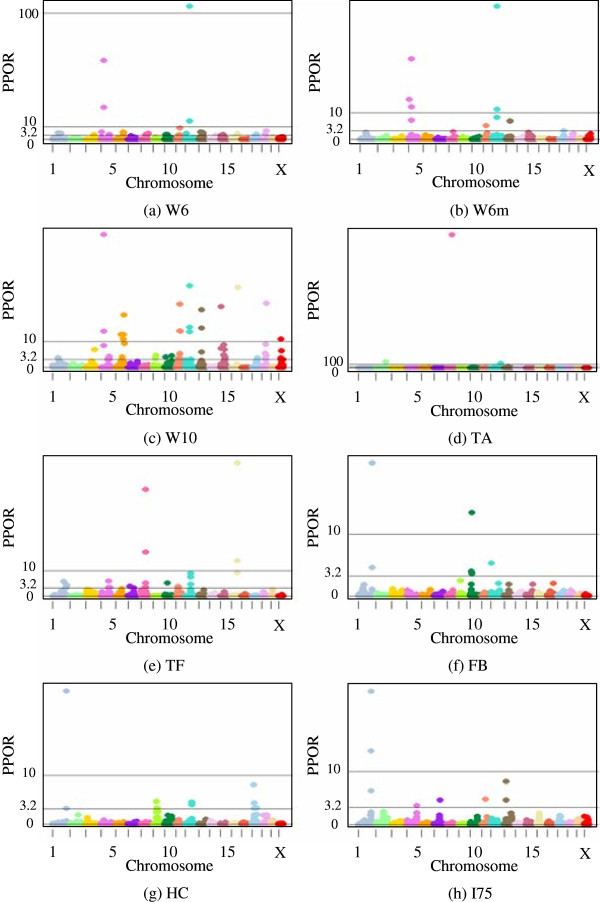
**Marker associations with QTLs based on model (2) using a 2.5% mixture model.** A. Weight at week 6 (W6), B. Weight at week 6 considering missing marker genotypes (W6m), C. Weight at week 10 (W10), D. Total activity in open field test (TA), E. Time freezing during cue (TF), F. Fecal boli after cue (FB), G. Hematocrit percentage (HC), H. Insulin level (I75). Changes in odds from prior to posterior probability (PPOR) of 3.2 < PPOR ≤ 10 denotes substantial evidence, 10 < PPOR ≤ 100 strong evidence and PPOR > 100 decisive evidence.

**Figure 3:**
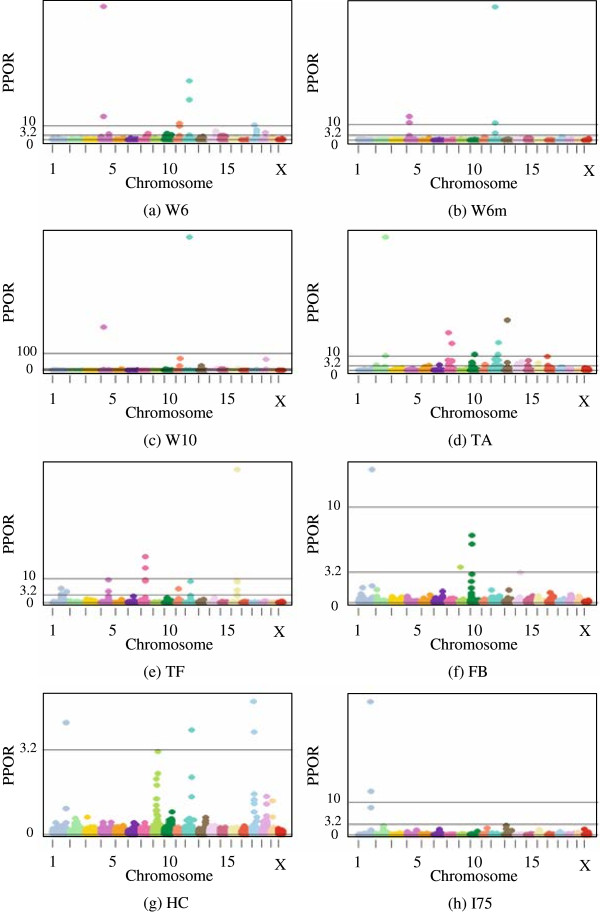
**Marker associations with QTLs based on model (3) using a 2.5% mixture model A.** Weight at week 6 (W6), **B.** Weight at week 6 considering missing marker genotypes (W6m), **C.** Weight at week 10 (W10), **D.** Total activity in open field test (TA), **E.** Time freezing during cue (TF), **F.** Fecal boli after cue (FB), **G.** Hematocrit percentage (HC), **H.** Insulin level (I75). Changes in odds from prior to posterior probability (PPOR) of 3.2 < PPOR ≤ 10 denotes substantial evidence, 10 < PPOR ≤ 100 strong evidence and PPOR > 100 decisive evidence.

As an illustration of the statistical relevance of particular markers, ratios of posterior to prior odds based on two 2.5% mixture models are shown in Table 6. Model (2) excludes and Model (3) includes polygenic effects. No trait showed markers with an increased evidence for an effect using mixture percentages higher than 10%. As an example of decreased number of markers showing evidence for an effect with increasing mixture percentages, the estimates for TA are illustrated in Figure 1. This pattern was found for all eight traits using both model (2) and model (3). Note that the number of SNP markers is not equal to the number of QTLs; a QTL effect may be spread over several markers in a region, whereby each individual marker picks up part of the effect of the QTL. The table lists the number of markers with substantial (3.2 < PPOR ≤ 10), strong (10 < PPOR ≤ 100) or decisive (PPOR > 100) effects. Moreover, Figures 2 and 3 show Manhattan plots of the PPOR per marker for model (2) and model (3), respectively. Generally model (2) detected more SNP markers to be associated with QTLs than model (3). Based on model (2), the two weight traits and TA showed the highest numbers of markers associated with QTLs (30 to 57 in total), followed by TF (21 in total). The three traits with the lowest heritabilities, FB, HC and I75, showed the lowest numbers of markers associated with QTLs (7 to 10 in total).

**Table 6:** Number of markers associated with QTLs classified by levels of evidence using 2.5% mixture model

	**Model (2)**			**Model (3)**		
**Trait**^**1**^	**Substantial**^**2**^	**Strong**^**3**^	**Decisive**^**4**^	**Substantial**^**2**^	**Strong**^**3**^	**Decisive**^**4**^
**W6**	26	3	1	12	6	0
**W6m**	5	5	0	1	5	0
**W10**	31	18	0	24	6	2
**TA**	41	13	3	39	8	0
**TF**	17	4	0	13	3	0
**FB**	5	2	0	3	1	0
**HC**	9	1	0	4	0	0
**I75**	6	2	0	1	2	0

1. Trait: W6 = Weight at week 6; W6m = Weight at week 6, missing marker genotypes were treated as 3^rd^ allele; W10 = Weight at week 10; TA = Total activity in open field test; TF = Time freezing during cue; FB = Fecal boli after cue; HC = Hematocrit percentage; I75 = Insulin level.

2. Substantial: Changes in odds from prior to posterior probability between >3.2 and ≤10.

3. Strong: Changes in odds from prior to posterior probability between >10 and ≤100.

4. Decisive: Changes in odds from prior to posterior probability >100.

In contrast to the variance estimates and PA, for which treating missing alleles as a separate 3^rd^ allele did not change their estimates, the number of markers with increased evidence to be associated with a QTL was much lower for W6m than for W6.

Figures 2 and 3 show that SNP markers, indicating the presence of QTLs, differed little for models (2) and (3) in terms of the location of the QTLs. Some variation was visible in the relative weight of markers located closely together, since markers situated near a QTL might each pick up part of the QTL effect. For example, for trait W6, in which a QTL region has been previously detected on chromosome 11, model (2) detected two adjacent markers in the region with a PPOR of 105 and 14, respectively (Figure 2), while the same markers for model (3) showed a PPOR of 29 and 42, respectively (Figure 3). Generally, model (2) detected more QTLs with decisive evidence except for W10, for which two decisive QTLs were found using model 3.

## Discussion

### Heritabilities

In general, higher heritabilities resulted in an increase in PA of genomic selection for all traits. Similar results were found for a different set of traits from this dataset [10,13], with PA as high as 0.67 for a trait with a high heritability (weight, h^2^ = 0.74), but as low as 0.27 for a trait with a low heritability (body length, h^2^ = 0.13). However, the relationship between heritability and PA was far from linear, as can be seen when comparing for example TF and I75, where the latter trait had a lower heritability but a higher PA using within family cross-validation. This might indicate that other factors besides the heritability have an influence on PA of a model.

### QTL and individual marker distribution

In addition to heritability, the influence of the number of QTLs on PA of a trait was also investigated. As pointed out earlier, the number of SNP markers to be associated with QTLs is higher than the number of QTLs so that we found more markers to have an substantial effect on QTLs than found by Valdar et al. [14]. Across traits, the number of markers associated with QTLs depended partially on the heritability, but especially for traits with low to moderate heritabilities the number of markers associated with QTLs varied substantially between traits with similar heritabilities.

There was a clear tendency for traits with fewer SNP markers associated with QTLs to have a lower PA in the case of between family cross-validation. The only exception was HC, which had the lowest PA but not the lowest total number of markers associated with QTLs. However, this trait had a relatively high number of markers classified with the lowest levels of evidence for QTLs compared to the other traits, which indicate their low effect size. For within family cross-validation the tendency was weaker.

Simulation studies, e.g. by Zhong et al. [15], Kizilkaya et al. [16] and Meuwissen and Goddard [17], have shown that the number of QTLs affecting a trait influences the performance of genomic selection, though the influence differed depending on the methodology that was used to estimate genomic breeding values. Kizilkaya et al. [16] found that for a given amount of genetic variance, an increase in the number of QTLs affecting a trait, and thereby a reduction of the variance attributed to a single QTL, led to a decrease in correlations between true and predicted genotype in both purebred (from 0.39 to 0.20) and multi-breed (from 0.42 to 0.30) situations. Assuming the availability of whole-genome sequence data, Meuwissen and Goddard [17] found that the accuracy of the predicted total genetic value using Bayesian methodology was higher in a scenario simulating three causative QTL per chromosome compared to that simulating 30 QTL. They suggested that the lower accuracy in the presence of more QTL may be caused by the fact that each QTL was associated with a smaller effect and therefore harder to detect and estimate accurately. For W6m, treating missing SNPs as a 3^rd^ allele reduced the number of detected SNPs associated with QTLs. This reduction had no influence on PA.

### Behavioural traits versus weight traits and physiological traits

Analysis of variance components based on models (2) and (3) indicated that behavioural traits showed in general much lower variability attributable to cage effects. The larger variability of cage effects for weight and physiological traits was also found by Valdar et al. [11] and various reasons, such as the more automated process used to record behavioural phenotypes, were discussed. Behavioural traits are generally difficult to collect in large quantities and difficult to measure directly, and therefore require suitable proxy traits.

### Cross-validation

There is a vast statistical literature on model comparison criteria using Bayesian and frequentist perspectives. In this work we focus on the use of genomic models to predict genetic values of individuals or to predict future observations. In such a context an obvious criterion of model comparison is their predictive ability. This was studied using cross-validation. Using this method, all three models performed equally well using within family cross-validation. Extensive pedigree information reduced the advantage of genomic information which provided only a small benefit relative to polygenic selection. However, with less close family ties, as is the case with between family cross-validation, genomic information became substantially more valuable, in agreement with other studies [13,18]. This effect was to some extent dependent on a number of factors discussed before, namely the heritability and number of QTLs affecting the trait. For FB and HC, two traits with low heritabilities and a small number of QTLs, genomic selection did not lead to an increase in PA. This indicates that a larger reference population is necessary for these traits to obtain more accurate inferences of genomic values as discussed by Goddard and Hayes [19]. For TF, a trait with moderate heritability and despite a low number of QTLs, genomic information led to a substantial increase in PA using between family cross-validation. For I75, a trait affected by a relatively large number QTLs, but with low heritability, inclusion of genomic information led to a moderate increase in PA when between family cross-validation was used.

### Inclusion of polygenic effects

Adding polygenic effects to a genomic model influenced the estimated variances by picking up the part of the genetic variance that was not captured by the genomic effects model. However it had little influence on PA. Legarra et al. [13] and De los Campos et al. [20] used the same dataset and found an increased PA using genomic information relative to polygenic information, but little difference between a solely genomic model and a combined genomic-polygenic model. A simulation study showed slight increases of accuracy when adding polygenic effects to the genomic model, but this was dependent on the extent of linkage disequilibrium between adjacent markers [21]. The same study also showed that a genomic model underestimates genetic variance, but that this is improved by adding a polygenic component, as was the case in this study.

### Influence of proportion of markers

A reduced number of markers assumed to have a substantial effect on the trait had an influence on estimates of variance but had no significant effect on PA for most traits. Mixture models explained less of the variance attributed to genomic effects, but resulted in better estimates of individual SNP effects. As a consequence, the PAs between models differed little. Within trait, there was a clear relationship between the mixture percentage and the number of SNP markers associated with QTLs, but no clear association with the PA. TA was the only trait to show a significant decrease in PA for within as well as between family cross-validation, but only in cases where the mixture proportion dropped below 7.5%. Weight traits showed almost the same trends for some mixture models, but for most traits no change in PA occurred even at a mixture proportion of 2.5%. Even though estimates for PA were not significantly different from each other, there seemed to be an optimum mixture percentage, with highest values obtained often around mixtures of 40%.

Su et al. [22] found similar results in dairy cattle when looking at the squared correlation between true and predicted breeding values in bulls, across a range of percentages of mixtures and traits. Reducing the percentages eventually led to lower correlations, but, depending on the trait, the decline was small and did not appear until percentages were below 20% (e.g. in the trait fat percentage in milk). In traits affected by a small number of QTLs with a large effect each, a larger part of the variance is accounted for by these QTLs. Reducing the proportion of SNPs might lead to an even higher proportion of variance explained by these QTLs and a more skewed distribution of SNP-effect size, as was shown by Su et al. [22, Figure 2. In contrast, in traits not affected by QTLs of large effect, the variance is shared more uniformly among all available SNPs. Similar to this link between SNP-effect size and mixture percentage, a larger number of markers showing a high PPOR and a more skewed distribution of PPOR was found in the present study when the mixture percentage was reduced. The relationship between mixture percentage and PPOR or SNP-effect size may be a reason for a slightly higher PA when the variance is distributed more evenly, which could be seen when comparing traits with more QTLs (e.g. TA) to traits with few QTLs (e.g. FB).

Due to the large costs of genotyping, low density SNP arrays or methodologies that reduce the numbers of animals to be genotyped are of great importance. Research in genome-wide association studies has found that a two-stage design with pre-selection of SNPs between steps can reduce costs substantially without reducing the power of the study [8,23]. Another strategy is the use of imputation of haplotypes or of missing genotypes, for example long-range phasing [24,25]. Our results indicate that, depending on trait characteristics such as heritability and number of QTLs involved, an optimum mixture percentage, i.e. an optimum number of SNPs considered to have a substantial effect, may exist. This indicated that a pre-selected, optimal subset of SNPs could be used for genomic selection of specific traits, where high efficiency is combined with lower financial costs. However, breeding programmes involve simultaneous selection of many traits, and depending on the degree of overlap of the selected SNP markers, the total number of selected SNPs may be considerably larger than the number of SNPs selected for a single trait.

## Conclusions

Genomic selection generally performed better than traditional polygenic selection, as indicated by an increase in PA. The increase in PA was most pronounced in the case of between family cross-validation. Larger increases in PA were found for traits with lower heritabilities, but the underlying number of QTLs affecting the trait had an important effect. Traits with a small number of QTLs showed lower PA using the genomic model compared to the polygenic model. Behavioural traits showed a lower variance of cage effects than other traits, but no difference in efficiency of genomic selection compared to traits with a similar heritability. Models including both polygenic and genomic effects captured more of the genetic variance, but did not improve PA. The dataset was restricted to genotyped animals only; incorporation of non-genotyped animals may show different results as a result of for example lower errors of estimation of fixed effects and higher accuracy of prediction of polygenic effects [26].

Reducing the number of SNP markers assumed to have a substantial effect in a mixture model did not significantly change PA for most traits, particularly in the context of within family cross-validation. The mixture approach showed that models using different percentages of SNPs affecting the trait performed efficiently even with low percentages, which may be of greater importance in the future with increasing sizes of SNP arrays.

In the present work, the a priori probability that a marker effect has a detectable effect was treated as a known parameter. In common with other results from the literature, this did not have a clear effect on the PA of the models. However as shown in Figure 1, the a priori probability influences the number of detectable markers a posteriori. Therefore when focus is on detection, it would be desirable to infer the probability of markers with detectable effects from the data. Recently, Bayesian implementations of such methods have been developed [27].

## Methods

### Animals

Data on 2,188 geno- and phenotyped mice provided by the Wellcome Trust Centre for Human Genetics were used to analyse the efficiency of genomic selection in seven traits. The data were freely available [28] and the care and use of animals were performed in compliance with the guidelines at the Wellcome Trust Centre for Human Genetics, University of Oxford, UK. The population has already been described and analyzed comprehensively in various papers including Solberg et al. [29] and Valdar et al. [11]. Therefore, only the aspects important for the present analysis will be highlighted here. Animals were obtained from crossing eight purebred mice strains, followed by 50 generations of pseudo-random mating. Data comprised of 175 full-sib families belonging to one generation and were collected over a period of three years, with a pedigree that consisted of parents and grandparents (2,890 animals in total). Parents and grandparents had no phenotypic records.

### Single nucleotide polymorphism markers

After removing uninformative markers, 10,496 SNPs were retained for the analysis. All animals had a call rate above 95% and 99% of all SNPs had call rates higher than 99%. Missing alleles were imputed at random based on the Hardy-Weinberg equilibrium conditional on the observed allelic frequencies of genotyped SNPs. The random numbers were generated based on a uniform distribution. The extent of linkage disequilibrium between pairs of markers was low with an r^2^ < 0.5 within 2 Mb and < 0.2 within 8 Mb [14] .

### Phenotypic data

Traits were chosen across a range of heritabilities, type (weight, behavioural or physiological) and number of QTLs (Table 7), based on Valdar et al. [11, 14, suppl.]. Weight traits included body weight at the start of the test at six weeks of age (W6) and body weight at the end of the test at ten weeks of age (W10). Behavioural traits included three measurements. One measurement was recorded as part of an open field test (a model of anxiety) at six weeks of age, namely total activity, measured as distance travelled in a time span of five minutes (TA). Two measurements were recorded as part of a cue conditioning test at seven weeks of age, whereby freezing to a tone after association with a foot shock was measured: time spent freezing during cue in minutes (TF) and number of fecal boli after cue (FB). Physiological traits were hematocrit percentage in blood as part of a full blood count test (HC) and insulin level at 75 minutes after intraperitoneal injection with glucose dose as part of a test to model type 2 diabetes mellitus, at nine weeks of age (I75). For further information regarding the biology behind these traits we refer to Solberg et al. [29]. These traits were normalized using the transformation given in Valdar et al. [11] and subsequently multiplied or divided by appropriate factors to avoid rounding errors in the multi-marker programme. To investigate the influence of low frequencies of missing SNPs, weight at 6 weeks was analysed with missing values for SNPs treated as a separate 3^rd^ allele with low frequency (W6m).

**Table 7:** Description of the traits used in the genetic analyses

**Trait**	**Type**	**Count**	**h**^**2;1**^	**QTL**^**1**^	**T**^**2**^
Weight at week 6 (W6)	Weight	1916	0.74	19	x^1/3^
Weight at week 10 (W10)	Weight	1880	0.62	20	x^1/3^
Total activity in open field test (TA)	Behavioural	1879	0.34	16	x
Time freezing during cue (TF)	Behavioural	1389	0.31	1	x
Fecal boli after cue (FB)	Behavioural	1511	0.10	2	(x + 1)^1/2^
Hematocrit percentage (HC)	Physiological	1578	0.11	1	x^3^
Insulin level^3^ (I75)	Physiological	1701	0.13	10	x^1/3^

1. h^2^; QTL: reported by Valdar et al. [11, 14, suppl.].

2. T: transformation used for the trait.

3. Insulin level: measured at 75 minutes after injection of glucose.

### Statistical analysis

All traits were treated as normally distributed and analyzed incorporating fixed effects and covariates based on the models reported by Valdar et al. [11]. Fixed effects were sex (W6, W6m, W10, TA, FB, HC, I75), year-month (W6, W6m, W10, TA), parity (W6, I75), experimenter (TA, I75), apparatus (TF) and month (I75); covariates comprised cage density (W6, W6m, W10, I75), age in days (W6, W6m, W10) and weight (HC, I75). Cage was added as a random effect for all traits. Cages consisted almost solely of animals from one family. For all practical purposes cage was nested within family (avg. 3.1 cages per family).

Three basic groups of models were used to compare changes in variance components and PA as a result of using genomic information. One model used only polygenic effects (1), a second model used only genomic effects (2), and a third model fitted both effects (3). For models (2) and (3), seven different sub-models were considered based on the percentage of markers that was assumed to have a substantial effect. This included a non-mixture model using 100% and six mixture models, ranging from 70%, 40%, 10%, 7.5%, 5% to 2.5% of the SNPs assumed to have a substantial effect. In the following, these sub-models will be labelled according to the mixture percentages. All analyses were performed using a Bayesian approach and implemented with Markov chain Monte Carlo methods [30] using the programme iBay [31]. The basic model using polygenic effects can be described as follows:

(1) $$y=\mu +X_{1}b+X_{2}c+Zu+e\text{,}$$

where *μ* fits a general mean and the vectors *b, c, u* and *e* fit thefixed,cage $\left(c∼N(0\text{,}\;I\sigma^{2}{}_{c})\right)$,polygenic $\left(u∼N(0\text{,}\;A\sigma^{2}{}_{u})\right)$ and residual effects $\left(e∼N(0\text{,}\;I\sigma^{2}{}_{e}\right)$, respectively. *I* is the identity matrix and *A* the additive genetic relationship matrix. *X*_*1*_*, X*_*2*_ and *Z* are incidence matrices relating the vectors *b, c* and *u* with *y*. This is the mixed model which is most commonly used to predict traditional breeding values in animal breeding programmes. For the model using genomic effects, model (1) was changed to a Bayesian multi-marker association model as follows:

(2) $$y=\mu +X_{1}b+X_{2}c+Qas+e\text{,}$$

where *Qas* fits the genomic effects, with *a* the vector representing effects associated with marker alleles $\left(a∼N\left(0,1\right)\right)$*s* a scaling factor modelling the variance explained by each marker and *Q* the design matrix linking alleles with markers [31]. Priors were assigned to the scaling factor *s* as follows for the non-mixture models:

(3) $$s∼TN_{>0}\left(0,\sigma^{2}{}_{g}\right)\text{,}$$

where $\sigma^{2}{}_{g}$ can be interpreted approximately as the expected average fitted variance per marker and *TN* denotes a truncated normal distribution. For mixture models the following scaling factors *s* were used:

(4) $$s∼\{\begin{array}{l} N\;\left(0,\sigma^{2}{}_{g0}\right)\;with\;probability\;\pi_{0}\\ TN_{>0}\left(0,\sigma^{2}{}_{g1}\right)\;with\;probability\;\pi_{1}=1-\pi_{0} \end{array}$$

where the first distribution models the markers with on average little to no effect at a proportion *π*_*0*_, and the second distribution models the markers that have a substantial effect at a proportion *π*_*1*_. The proportions of markers *π*_*1*_ were varied across mixture models ranging from 100 to 2.5%. Variances for the first distribution $\left(\sigma^{2}{}_{g0}\right)$ were set to 1% of the phenotypic variance of the trait divided by the number of markers. No polygenic effects were present and all other effects were as described for model (1). Using the methodology of genomic selection as described by Meuwissen et al. [1], it was possible to solve models with more markers than phenotypic records. The last model, which combined both genetics effects of model (1) and (2), can be as described as follows:

(5) $$y=\mu +X_{1}b+X_{2}c+Qas+Zu+e\text{,}$$

where the effects are as defined earlier. Here the polygenic variance of *u* accounts for genetic variation which could not be explained by the genomic markers *a*.

### Variance components

Estimates for the variance of polygenic effects $\left(\sigma^{2}{}_{u}\right)$, variance of genomic effects $\left(\sigma^{2}{}_{a}\right)$, cage variance $\left(\sigma^{2}{}_{c}\right)$, residual variance $\left(\sigma^{2}{}_{e}\right)$ and total phenotypic variance $\left(\sigma^{2}{}_{p}\right)$ were calculated using information from all animals that had both genomic and phenotypic information. The variance of genomic effects $\sigma^{2}{}_{a}$ is calculated as the sum of the contributions to the genetic variance from each marker, plus all possible covariances due to linkage disequilibrium, taking into account the allele frequencies. The heritabilities for the polygenic effects $\left({\text{h}}^{2}{}_{\text{u}}\right)$ and genomic effects $\left({\text{h}}^{2}{}_{\text{a}}\right)$ were calculated based on their corresponding variance components $(\sigma^{2}{}_{u}\;\text{and}\;\sigma^{2}{}_{a}$, respectively) as proportion of the phenotypic variance. The software iBay required that animals had both genomic and phenotypic data available to be included in the analysis.

### Predictive ability

PA was calculated as the Pearson’s correlation between a predicted observation and the corresponding realized observation. Realized observation was calculated as the phenotype corrected for fixed effects and covariates, while the predicted observation was the estimated breeding value, as was done by Legarra et al. [13]. To predict these observations, a cross validation approach was used, whereby the dataset was split into a validation set and a training set. The validation set contained the animals for which the observation had to be predicted, while the training set was used to estimate the parameters for the prediction model. Size of the training set is of importance for the estimation of accurate breeding values [19] and to ensure a sufficient size of training population, a 1:5 proportion of validation to training dataset was used. Only animals from families with at least two members were used to create validation sets (~ 80% of all animals). These animals were randomly split into five groups to create five validation sets. Thus each validation set contained ~16% of all animals. This was repeated to create ten validation sets in total. Each validation set had a corresponding training set, which contained the remaining animals with phenotypic data.

Two different routines for splitting the data were used: within family and between family cross-validation. For within family cross-validation, full sib families were randomly split between training and validation set such that each set contained at least one animal from a family. For between family cross-validation, families were split such that no full sib family would have animals in both datasets simultaneously. As a result, for between family cross-validation no close genetic connectedness due to full sib families was available between training and validation data. In the case of within family cross-validation, full sibs with phenotypic data linked the breeding values of the training and validation data.

### Importance of individual markers

As an illustration, the relative importance of individual markers was quantified via the computation of Bayes Factors, conditional on either model (2) or model (3). The correct inferences about the statistical relevance of particular markers could involve, first, calculation of the posterior probability of each model. Secondly one could report Bayes factors conditional on the model with largest posterior probability, or averaging over all models. This task was judged to be computationally too burdensome and was not undertaken in this study. As indicated in Table 7, traits were chosen across a range of number of QTLs, ranging from as low as 1 for TF and HC up to 20 for W10. The objective was to compare the performance of genomic models (2) and (3) in finding regions with evidence of a marker having an increased effect, and to study how the number of QTLs affecting a trait influences the efficiency of genomic selection. Using the Bayesian approach implemented in the programme iBay [31], the Bayes Factor computed as the change in prior to posterior odds (PPOR) for each marker was calculated with the following formula:

(6) $$\text{PPOR }=({\hat{p}}_{1}/(1-{\hat{p}}_{1}))/\left(\pi_{1}/1-\pi_{1}\right)\text{,}$$

where ${\hat{p}}_{1}$is the estimate for the posterior probability of the marker having a substantial effect, and *π*_*1*_ the a priori probability that the marker has a substantial effect. Results were plotted per trait for all markers, whereby a PPOR > 3.2 can be interpreted as substantial evidence for the marker to have an increased effect, a PPOR > 10 as strong evidence, and a PPOR > 100 as decisive [31].

## Competing interests

The authors have no competing interests.

## Authors’ contributions

DK conducted the statistical analyses of the data, discussed the interpretation of the results and prepared and drafted the manuscript. DS suggested the use of mixture models, helped with the interpretation of the results and contributed to the final manuscript. GS assisted with the statistical analysis and provided interpretations of the results. LJ provided assistance to use the software for the statistical analysis and provided the idea how missing genotypes should be fitted. CA helped with the interpretation of the results and the writing of the manuscript. RR coordinated the research, initiated the project, identified the data and its analysis and helped with the interpretation of the results and writing of the manuscript. All authors read and approved the final manuscript.

## References

[B1] Meuwissen THE Hayes BJ Goddard ME Prediction of total genetic value using genome-wide dense marker maps Genetics 2001 157 4 1819 1829 11290733 10.1093/genetics/157.4.1819 PMC1461589

[B2] Wong CK Bernardo R Genomewide selection in oil palm: increasing selection gain per unit time and cost with small populations Theor Appl Genet 2008 116 6 815 824 10.1007/s00122-008-0715-5 18219476

[B3] Schaeffer LR Strategy for applying genome-wide selection in dairy cattle J Anim Breed Genet 2006 123 4 218 223 10.1111/j.1439-0388.2006.00595.x 16882088

[B4] Turner SP Roehe R D'Eath RB Ison SH Farish M Jack MC Lundeheim N Rydhmer L Lawrence AB Genetic validation of postmixing skin injuries in pigs as an indicator of aggressiveness and the relationship with injuries under more stable social conditions J Anim Sci 2009 87 10 3076 3082 10.2527/jas.2008-1558 19574573

[B5] Visscher PM Macgregor S Benyamin B Zhu G Gordon S Medland S Hill WG Hottenga JJ Willemsen G Boomsma DI Genome partitioning of genetic variation for height from 11,214 sibling pairs Am J Hum Genet 2007 81 5 1104 1110 10.1086/522934 17924350 PMC2265649

[B6] Manolio TA Collins FS Cox NJ Goldstein DB Hindorff LA Hunter DJ McCarthy MI Ramos EM Cardon LR Chakravarti A Finding the missing heritability of complex diseases Nature 2009 461 7265 747 753 10.1038/nature08494 19812666 PMC2831613

[B7] Olsen HG Hayes BJ Kent MP Nome T Svendsen M Lien S A genome wide association study for QTL affecting direct and maternal effects of stillbirth and dystocia in cattle Anim Genet 2010 41 3 273 280 10.1111/j.1365-2052.2009.01998.x 19968646

[B8] Li J Prioritize and select SNPs for association studies with multi-stage designs J Comput Biol 2008 15 3 241 257 10.1089/cmb.2007.0090 18352819 PMC3326652

[B9] Solberg TR Sonesson AK Woolliams JA Meuwissen THE Reducing dimensionality for prediction of genome-wide breeding values Genet Sel Evol 2009 41 8 10.1186/1297-9686-41-8 19296851 PMC2671482

[B10] Usai MG Goddard ME Hayes BJ LASSO with cross-validation for genomic selection Genet Res 2009 91 6 427 436 10.1017/S0016672309990334 20122298

[B11] Valdar W Solberg LC Gauguier D Cookson WO Rawlins JNP Mott R Flint J Genetic and environmental effects on complex traits in mice Genetics 2006 174 2 959 984 10.1534/genetics.106.060004 16888333 PMC1602068

[B12] Yang JA Benyamin B McEvoy BP Gordon S Henders AK Nyholt DR Madden PA Heath AC Martin NG Montgomery GW Common SNPs explain a large proportion of the heritability for human height Nature Genet 2010 42 7 565 U131 10.1038/ng.608 20562875 PMC3232052

[B13] Legarra A Robert-Granie C Manfredi E Elsen J-M Performance of genomic selection in mice Genetics 2008 180 1 611 618 10.1534/genetics.108.088575 18757934 PMC2535710

[B14] Valdar W Solberg LC Gauguier D Burnett S Klenerman P OCookson W Taylor MS Rawlins JNP Mott R Flint J Genome-wide genetic association of complex traits in heterogeneous stock mice Nature Genet 2006 38 8 879 887 10.1038/ng1840 16832355

[B15] Zhong SQ Dekkers JCM Fernando RL Jannink JL Factors affecting accuracy from genomic selection in populations derived from multiple inbred lines: a barley case study Genetics 2009 182 1 355 364 10.1534/genetics.108.098277 19299342 PMC2674832

[B16] Kizilkaya K Fernando RL Garrick DJ Genomic prediction of simulated multibreed and purebred performance using observed fifty thousand single nucleotide polymorphism genotypes J Anim Sci 2009 88 2 544 551 19820059 10.2527/jas.2009-2064

[B17] Meuwissen T Goddard M Accurate Prediction of Genetic Values for Complex Traits by Whole-Genome Resequencing Genetics 2010 185 2 623 631 10.1534/genetics.110.116590 20308278 PMC2881142

[B18] Lee SH Van der Werf JHJ Hayes BJ Goddard ME Visscher PM Predicting unobserved phenotypes for complex traits from whole-genome SNP data PLoS Genet 2008 4 10 11 10.1371/journal.pgen.1000231 PMC2565502 18949033

[B19] Goddard ME Hayes BJ Mapping genes for complex traits in domestic animals and their use in breeding programmes Nat Rev Genet 2009 10 6 381 391 10.1038/nrg2575 19448663

[B20] Delos Campos G Naya H Gianola D Crossa J Legarra A Manfredi E Weigel K Cotes JM Predicting quantitative traits with regression models for dense molecular markers and pedigree Genetics 2009 182(375 385 19293140 10.1534/genetics.109.101501 PMC2674834

[B21] Calus MPL Veerkamp RF Accuracy of breeding values when using and ignoring the polygenic effect in genomic breeding value estimation with a marker density of one SNP per cM J Anim Breed Genet 2007 124 6 362 368 10.1111/j.1439-0388.2007.00691.x 18076473

[B22] Su G Guldbrandtsen B Gregersen VR Lund MS Preliminary investigation on reliability of genomic estimated breeding values in the Danish Holstein population J Dairy Sci 2009 93 3 1175 1183 20172238 10.3168/jds.2009-2192

[B23] Satagopan JM Elston RC Optimal two-stage genotyping in population-based association studies Genet Epidemiol 2003 25 2 149 157 10.1002/gepi.10260 12916023 PMC8978311

[B24] Daetwyler HD Wiggans GR Hayes BJ Woolliams JA Goddard ME Imputation of missing genotypes from sparse to high density using long-range phasing9th World Congress on Genetics Applied to Livestock Production, Leipzig, Germany: 2010 2010, Communication No. 0539 10.1534/genetics.111.128082 PMC3176129 21705746

[B25] Hickey JM Kinghorn BP Cleveland MA Tier B Van der Werf JHJ Recursive long range phasing and long haplotype library imputation: building a global haplotype library for Holstein cattle9th World Congress on Genetics Applied to Livestock Production, Leipzig, Germany: 2010 2010 Communication No. 0934

[B26] Christensen OF Lund MS Genomic prediction when some animals are not genotyped Genet Sel Evol 2010 42 8 10.1186/1297-9686-42-8 20105297 PMC2834608

[B27] Habier D Fernando RL Kizilkaya K Garrick DJ Extension of the bayesian alphabet for genomic selection BMC Bioinformatics 2011 12 186 10.1186/1471-2105-12-186 21605355 PMC3144464

[B28] The Genetic Architecture of Complex Traits in Heterogeneous Stock Mice http://gscan.well.ox.ac.uk/

[B29] Solberg LC Valdar W Gauguier D Nunez G Taylor A Burnett S Arboledas-Hita C Hernandez-Pliego P Davidson S Burns P A protocol for high-throughput phenotyping, suitable for quantitative trait analysis in mice Mamm Genome 2006 17 2 129 146 10.1007/s00335-005-0112-1 16465593

[B30] Sorensen D Gianola D Likelihood Bayesian and MCMC methods in quantitative genetics 2002 Springer Verlag

[B31] Janss LLG iBay manual version 1.46 2008 Janss Biostatistics, Leiden, The Netherlands

